# Supplementary material for: Predicting materials properties without crystal structure: deep representation learning from stoichiometry
Source: Nat Commun. 2020 Dec 8;11:6280. doi: 10.1038/s41467-020-19964-7 (PMC7722901; doi:10.1038/s41467-020-19964-7)
Supplement: Supplementary file 1 — Supplementary Information [file 41467_2020_19964_MOESM1_ESM.pdf]

Supplementary Information:
Predicting materials properties without crystal structure:
Deep representation learning from stoichiometry

Rhys E. A. Goodall and Alpha A. Lee*
University of Cambridge, Cavendish Laboratory, Cambridge, UK

SUPPLEMENTARY NOTE 1: MISCALIBRATION OF UNCERTAINTIES

Uncertainty estimates produced using the *Deep Ensemble* approach [1] for regression tasks are typically not calibrated to the magnitude of the error out-of-the-box (as shown in Supplementary Figure 4). However, depending on the application post-hoc calibration approaches can be applied to correct for this [2–4]. Fortunately, within materials discovery uncertainty estimates are typically made use of via active learning workflows [5–8] that are robust against miscalibration of this sort. In active learning workflows an acquisition function is used to select candidates or batches of candidates to test. For many choices of acquisition function [9] the acquisition function contains hyperparameters that allow for the exploration-exploitation trade-off of the search process to be tuned. Often selecting such hyperparameters is akin to adjusting the temperature of the uncertainty distribution i.e. equivalent to a post-hoc calibration of the uncertainty. When using such an acquisition function, as selecting hyperparameters is non-trivial, even for calibrated uncertainties, the miscalibration of the model uncertainties is therefore not prohibitive for materials discovery workflows provided the uncertainty estimates are well-behaved despite potential miscalibration. Indeed, due to the difficulty of selecting good hyperparameters, search strategies that acquire batches of points by selecting multiple candidates under a range of hyperparameters have been proposed for effectively searching chemical spaces [10].

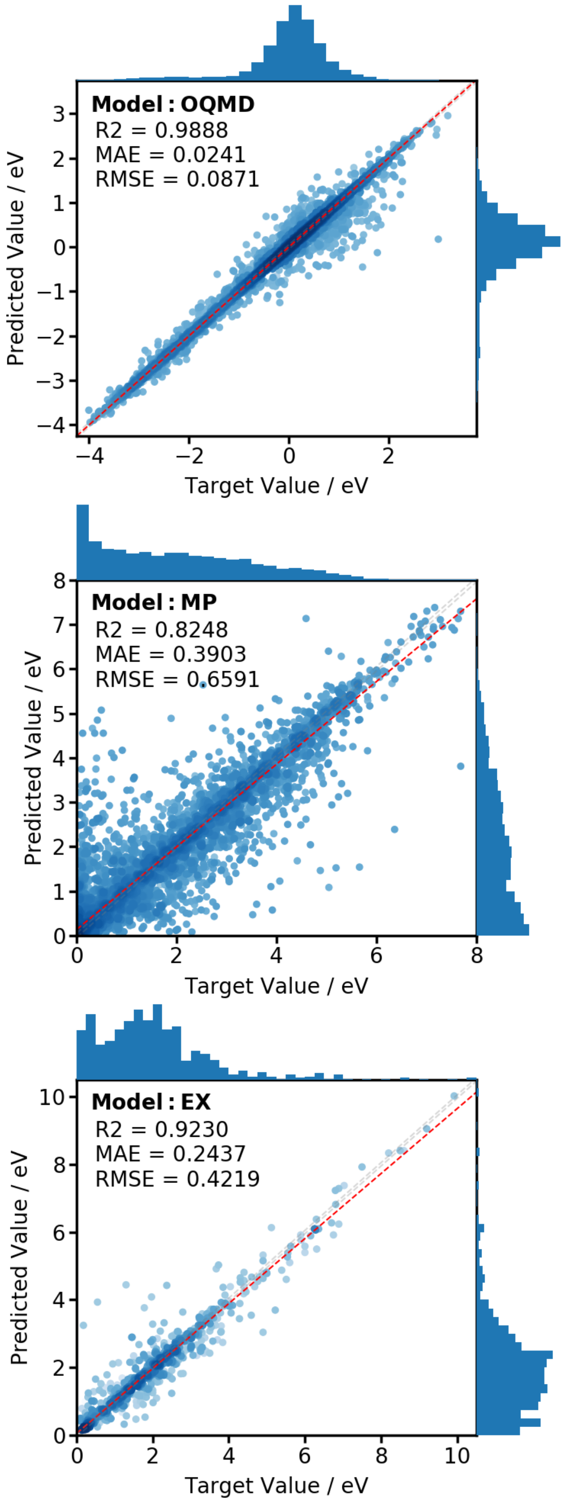

Supplementary Figure 1. **Prediction Scatter Plots for Test Sets.** The figure shows scatter plots of with marginal histograms for the model predictions on the test sets for the OQMD, MP and EX data sets. The points are shaded according their log density with dense regions of the scatter plot being darker. Both the marginal histograms are plotted with the same scale for the count such that they can be compared fairly. The red dotted line shows a robust (Huber) line of best fit for the data.

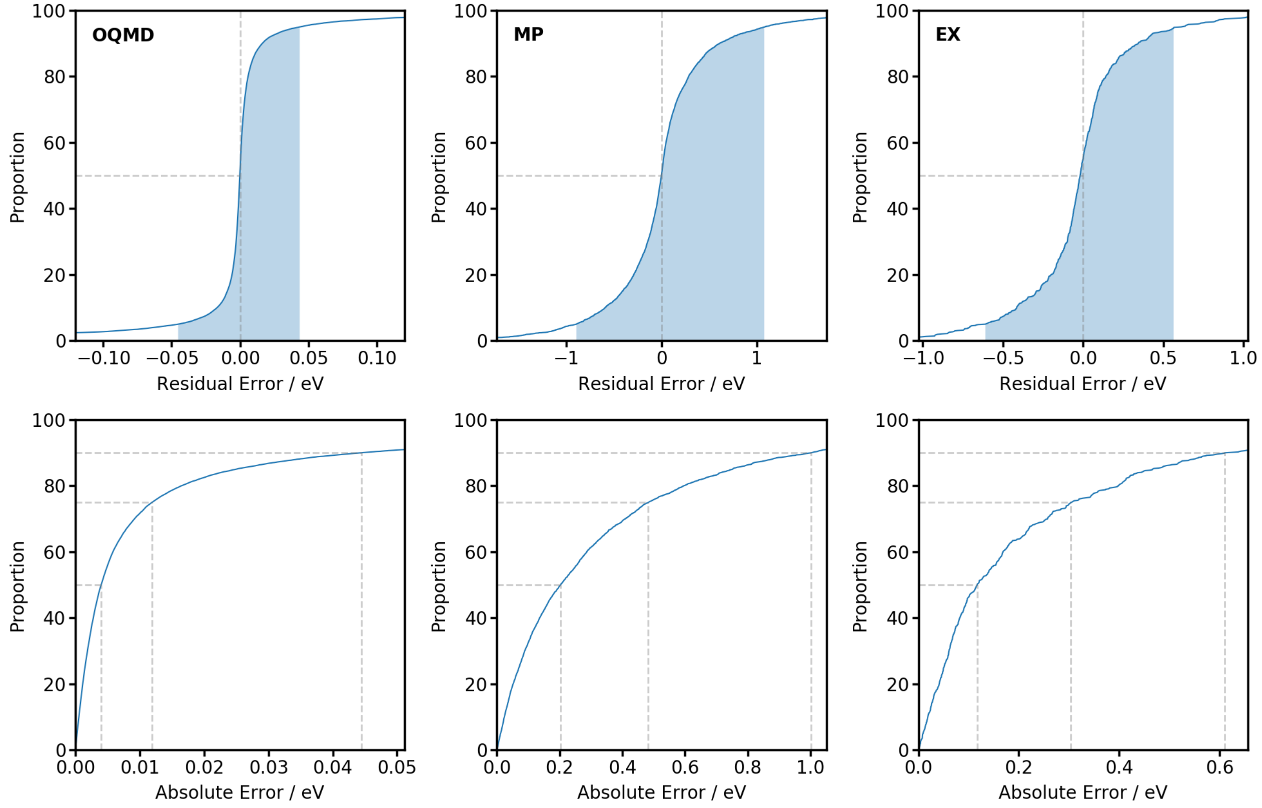

Supplementary Figure 2. **Cumulative Error Distributions on Test Sets.** The figure shows cumulative distributions for the residuals and absolute error for the model predictions on the test sets for the OQMD, MP and EX data sets. In the top row guidelines are shown for zero error and 50% of the data. The blue shaded region shows highlights the region in which 90% of the data falls. In the second plot guidelines are shown for 50%, 75% and 90% of the data.

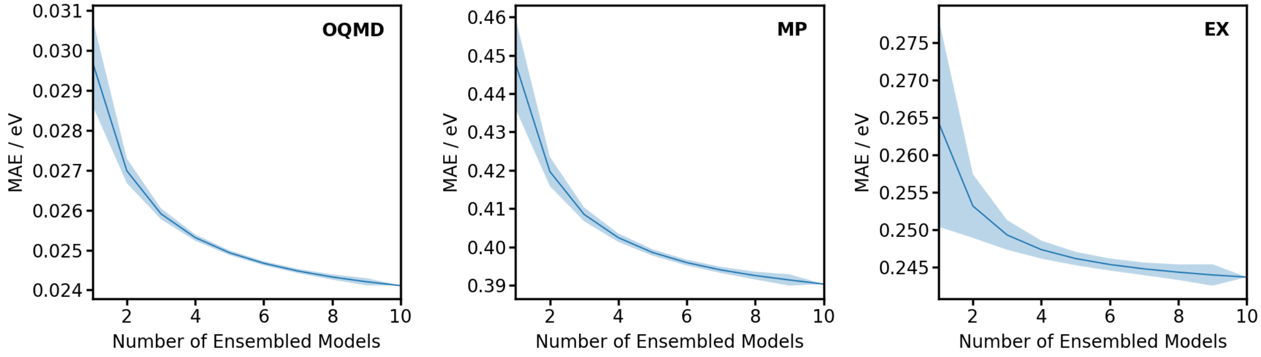

Supplementary Figure 3. **Ensemble Improvement with Additional Models.** The figure shows how the MAE decays as additional models are added to the ensemble. The curve is calculated by considering all possible combinations of available single models. The shaded region indicates the 5 times the standard error of the different combinations to give an illustration of the variability of different ensembles.

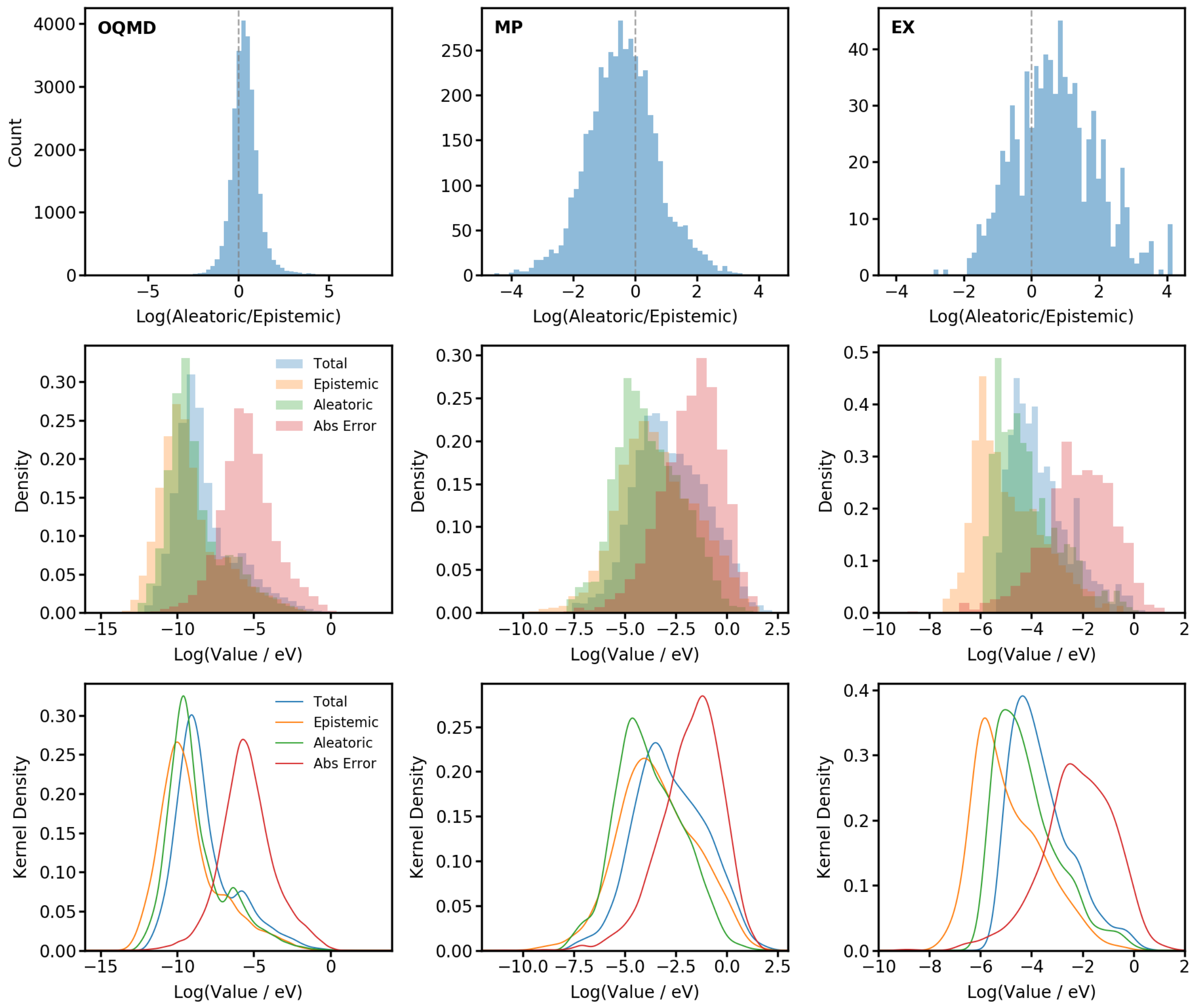

Supplementary Figure 4. **Uncertainty Distributions on Test Sets.** The figure explores the distributions of the different components of the predictive uncertainties for the OQMD, MP and EX data sets. The first row shows histograms of the log ratio of the aleatoric and epistemic contributions to the uncertainty. In each instance we see relatively symmetrical distributions but the magnitudes of the two contributions are not equal this is apparent in the fact that these distributions are not centred around 0. The second row shows histograms for how the uncertainty estimates (Aleatoric, Epistemic and Total i.e. Aleatoric + Epistemic) and absolute residuals (Error) are distributed. Critically we see that the uncertainty estimates produced by the model are mis-calibrated. The third row is equivalent to the second row but uses a kernel density function to allow the distributions to be plotted as curves for improved clarity.

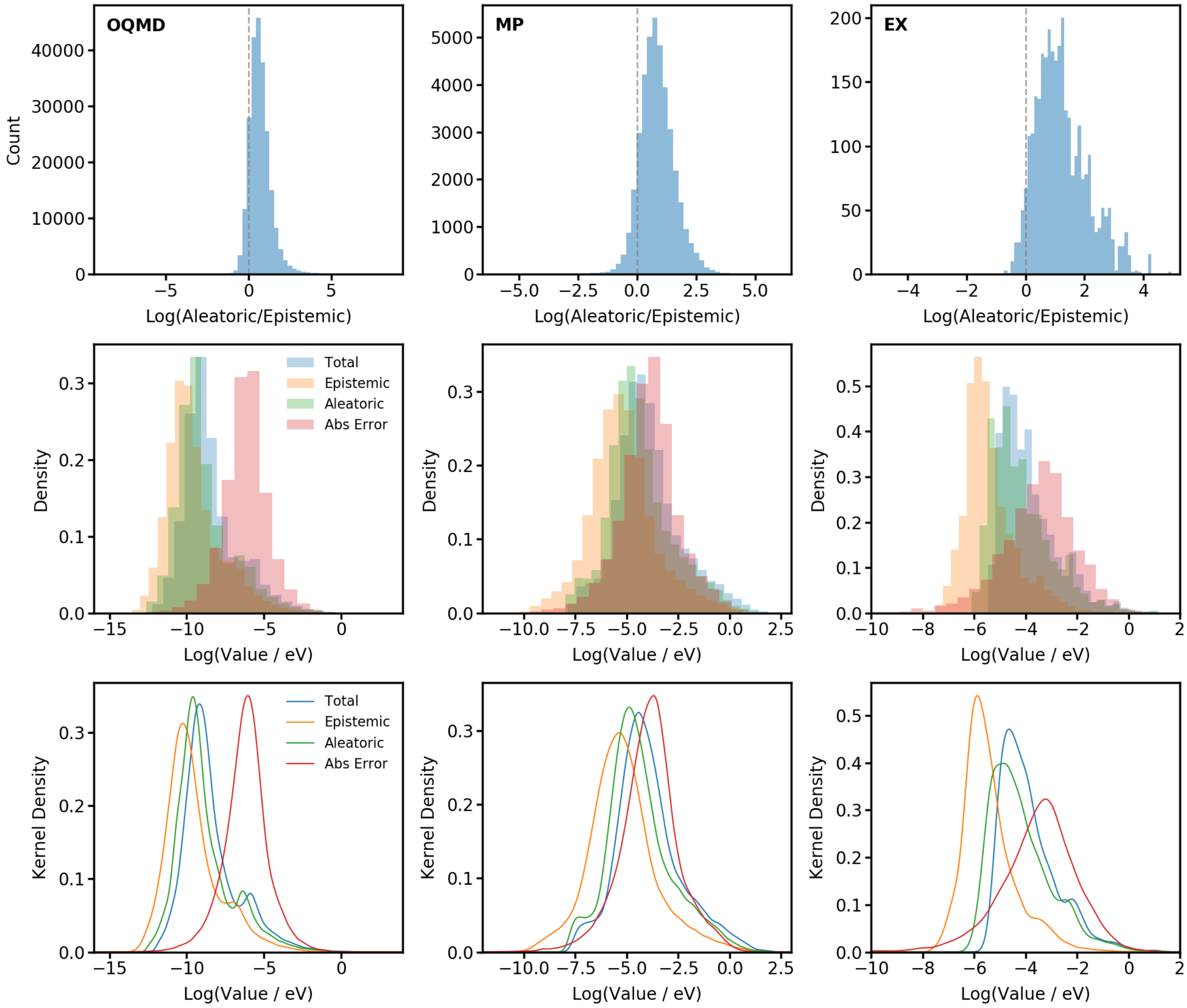

Supplementary Figure 5. **Uncertainty Distributions on Training Sets.** A repeat of the analysis in Supplementary Figure 4. on the training sets rather than the test sets. In the first row we see that in the training set the epistemic uncertainty is lower. This is observed as positive offset of the log ratio of the aleatoric and epistemic contributions to the uncertainty. This behaviour is consistent with the expectation that within the training set all the different models in the ensemble should agree strongly on their predictions, and therefore, result in a low epistemic uncertainty. The second and third rows show that in general the predicted uncertainties are also mis-calibrated on the training set.

* Correspondence email address: aal44@cam.ac.uk

[1] B. Lakshminarayanan, A. Pritzel, and C. Blundell, in *Advances in Neural Information Processing Systems* (2017) pp. 6402–6413.
[2] C. Guo, G. Pleiss, Y. Sun, and K. Q. Weinberger, in *International Conference on Machine Learning* (2017) pp. 1321–1330.
[3] H. Song, T. Diethe, M. Kull, and P. Flach, in *International Conference on Machine Learning* (2019) pp. 5897–5906.
[4] Y. Ovadia, E. Fertig, J. Ren, Z. Nado, D. Sculley, S. Nowozin, J. Dillon, B. Lakshminarayanan, and J. Snoek, in *Advances in Neural Information Processing Systems* (2019) pp. 13991–14002.
[5] B. Meredig, E. Antono, C. Church, M. Hutchinson, J. Ling, S. Paradiso, B. Blaiszik, I. Foster, B. Gibbons, J. Hattrick-Simpers, *et al.*, Molecular Systems Design & Engineering **3**, 819 (2018).
[6] L. Bassman, P. Rajak, R. K. Kalia, A. Nakano, F. Sha, J. Sun, D. J. Singh, M. Aykol, P. Huck, K. Persson, *et al.*, npj Computational Materials **4**, 1 (2018).
[7] A. Solomou, G. Zhao, S. Boluki, J. K. Joy, X. Qian, I. Karaman, R. Arróyave, and D. C. Lagoudas, Materials & Design **160**, 810 (2018).
[8] T. Lookman, P. V. Balachandran, D. Xue, and R. Yuan, npj Computational Materials **5**, 1 (2019).
[9] A. Agnihotri and N. Batra, Distill 10.23915/distill.00026 (2020).
[10] F. Hase, L. M. Roch, C. Kreisbeck, and A. Aspuru-Guzik, ACS central science **4**, 1134 (2018).
